# Mitochondrial DNA and Exercise: Implications for Health and Injuries in Sports

**DOI:** 10.3390/cells10102575

**Published:** 2021-09-28

**Authors:** Giada Zanini, Anna De Gaetano, Valentina Selleri, Gustavo Savino, Andrea Cossarizza, Marcello Pinti, Anna Vittoria Mattioli, Milena Nasi

**Affiliations:** 1 Department of Life Sciences, University of Modena and Reggio Emilia, 41125 Modena, Italy; giada.zanini@unimore.it (G.Z.); anna.degaetano@unimore.it (A.D.G.); valentina.selleri@unimore.it (V.S.); marcello.pinti@unimore.it (M.P.); 2 National Institute for Cardiovascular Research-INRC, 40126 Bologna, Italy; andrea.cossarizza@unimore.it (A.C.); vittoria@unimore.it (A.V.M.); 3 Department of Public Healthcare, Sports Medicine Service, Azienda USL of Modena, 41121 Modena, Italy; g.savino@ausl.mo.it; 4 Department of Medical and Surgical Sciences for Children and Adults, University of Modena and Reggio Emilia, 41125 Modena, Italy; 5 Department of Surgery, Medicine, Dentistry and Morphological Sciences, University of Modena and Reggio Emilia, 41125 Modena, Italy

**Keywords:** cf-mtDNA, trauma, sport, exercise, NET

## Abstract

Recently, several studies have highlighted the tight connection between mitochondria and physical activity. Mitochondrial functions are important in high-demanding metabolic activities, such as endurance sports. Moreover, regular training positively affects metabolic health by increasing mitochondrial oxidative capacity and regulating glucose metabolism. Exercise could have multiple effects, also on the mitochondrial DNA (mtDNA) and vice versa; some studies have investigated how mtDNA polymorphisms can affect the performance of general athletes and mtDNA haplogroups seem to be related to the performance of elite endurance athletes. Along with several stimuli, including pathogens, stress, trauma, and reactive oxygen species, acute and intense exercise also seem to be responsible for mtDNA release into the cytoplasm and extracellular space, leading to the activation of the innate immune response. In addition, several sports are characterized by a higher frequency of injuries, including cranial trauma, associated with neurological consequences. However, with regular exercise, circulating cell-free mtDNA levels are kept low, perhaps promoting cf-mtDNA removal, acting as a protective factor against inflammation.

## 1. Introduction

Mitochondria are cellular organelles involved in various biological processes, such as energy production, metabolism, cell death, and inflammation. Recently, several studies have highlighted the role of mitochondria and their genome, the mitochondrial DNA (mtDNA), in the response to exercise and physical activity, with a growing interest in the implications in elite/professional sports. Mitochondria and their metabolism are crucial for the systemic adaptive response, including to exercise, which represents a powerful tool for the prevention of several pathologies [1]. Oxygen uptake and its mitochondrial consumption influences exercise capacity, modulating skeletal muscle activity. Regular exercise improves mitochondrial energy production for muscle contractile activity sustention [2]. MtDNA is involved in response to exercise in its intra- and extracellular forms; it has been shown that the presence of different mtDNA haplogroups influence the performance of elite endurance athletes [3]. Moreover, when mtDNA is released from mitochondria, a significant pro-inflammatory cellular response is induced. The presence of hypomethylated CpG islands, similar to bacterial ones, or aberrant CpG methylation motifs are recognized as “non-self” by the innate immune system [4,5,6] and, when extracellularly released, act as a mitochondrial damage-associated molecular patterns (mDAMPs) [7]. Along with several stimuli, including pathogens, stress, trauma, and reactive oxygen species (ROS), acute and intense exercise also seem responsible for mtDNA release into the cytoplasm and extracellular space, leading to activation of the innate immune response. Several sports are characterized by a higher frequency of injury/trauma, including cerebral injury. These sports may contribute to an increase in circulating cell-free (cf) mtDNA in the systemic circulation or the central nervous system, contributing at least in part to the systemic and/or neuro-inflammation, associated with neurological damage. However, with regular exercise, cf-mtDNA levels are kept low, perhaps promoting cf-mtDNA removal, and therefore acting as a protective factor against inflammation [8,9]. Thus, the release of cf-mtDNA during physical activities or sports could be considered a double-edged sword.

In this review, we discuss the main findings related to the effects of cf-mtDNA in sport, with particular attention to the mechanisms underpinning these effects.

## 2. Role of Mitochondrial Metabolism and mtDNA Haplogroups on Exercise

**Figure 1:**
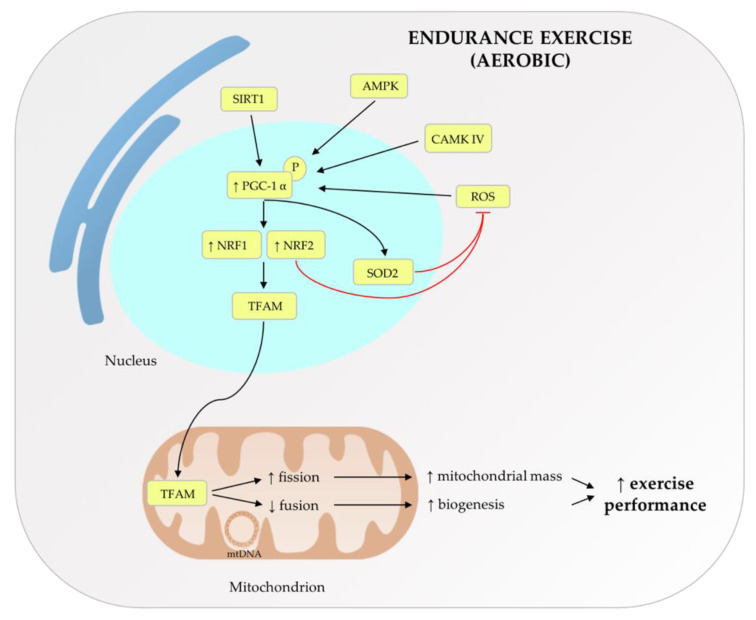
The main changes in cellular and mitochondrial factors, induced by aerobic, endurance exercise contribute to the exercise performance improvement.

Genetic and epigenetic factors may modulate adaptive responses to exercise through different mechanisms. Oxygen uptake and its mitochondrial utilization influence exercise capacity—the maximum individual tolerance to physical effort. In response to acute exercise, in skeletal muscle, the transcription cofactor PGC-1α can be activated by silent mating type information regulation 2 homolog 1 (SIRT1), calcineurin A, Ca^2+^/calmodulin-dependent protein kinase IV, AMP-activated protein kinase (AMPK), and ROS [10]. PGC-1α then induces the activation of nuclear respiratory factor-1 (NRF1) and -2 (NRF2). NRF1 and NRF2 stimulate mitochondrial transcription factor A (TFAM), which translocates into the mitochondria and initiates the replication and transcription of mtDNA [11]. This process promotes the proper formation of electron transport chain multisubunit complexes and an increment of mitochondrial metabolic capacity [10]. In addition, PGC-1α can control ROS levels, which increase during exercise, thanks to the binding between NRF2 and antioxidant response elements (ARE) [12]. PGC-1α has a role in the regulation of mitochondrial antioxidant activity, also inducing superoxide dismutase 2 (SOD2) expression after exercise, counteracting SOD2 physiological age-associated decline [13]. During aging, there is also dysregulation of mitochondrial dynamics, fusion, and fission in skeletal muscle, leading to pervasive alterations of mitochondrial functions, such as ROS production, respiratory capacity, coupling, and apoptotic sensitivity [1]. With exercise training, the differences between young and aged muscles are decreased in terms of the levels of various mitochondrial markers, such as mitofusin 1 (Mfn1) and 2 (Mfn2), NRF1, Dynamin-like 120 kDa protein (OPA-1), Fis1, TFAM, and dynamin-related protein 1 (DRP-1) [9]. Thus, regular exercise could be useful for counteracting age-related changes observed in muscle mitochondria [9]. Sirtuins (SIRTs), a family of NAD-dependent deacetylase including seven members with different cellular locations, play a particularly important role in metabolic adaptation induced by regular exercise. SIRT’s role is critical in caloric restriction adaptation, aging and exercise, through the control of vital cellular signaling pathways [14]. In organs with a higher metabolic rate, such as the kidney, heart, and liver, exercise training and caloric restriction up-regulate the levels of SIRT3, a sirtuin located in the mitochondrial matrix [15,16]. Hence, trained subjects have elevated SIRT3 levels in skeletal muscle [17]. A decline in the amount of skeletal muscle mtDNA copy numbers and SIRT3 in addition to higher acetylation of mitochondrial proteins has been observed in the aging of both women [18] and men [19]. Decreased mortality and beneficial effects on organ functions, including upregulation of molecular pathways for the protection against ROS, are correlated with an elevation of maximum oxygen uptake (VO_2_max), a well-studied marker of physical fitness, during exercise [20]. In cell culture, oxidative stress causes the translocation of MOTS-c, a mitochondrial-derived peptide, to the nucleus, promoting an antioxidant response. Modulation of MOTS-c levels seems to be AMPK-dependent [21]. An increment of MOTS-c in plasma and muscle has been reported in healthy young men after acute high-intensity cycling exercise, improving whole-body energy metabolism [22,23]. Figure 1 summarizes the main changes in cellular and mitochondrial factors induced by aerobic endurance exercise which contribute to the increment of exercise performance. While one session of aerobic exercise is not enough to cause mtDNA deletion in rat skeletal muscle, a heavy aerobic training session can cause mtDNA alterations that in turn could lead to cell damage [24].

**Figure 2:**
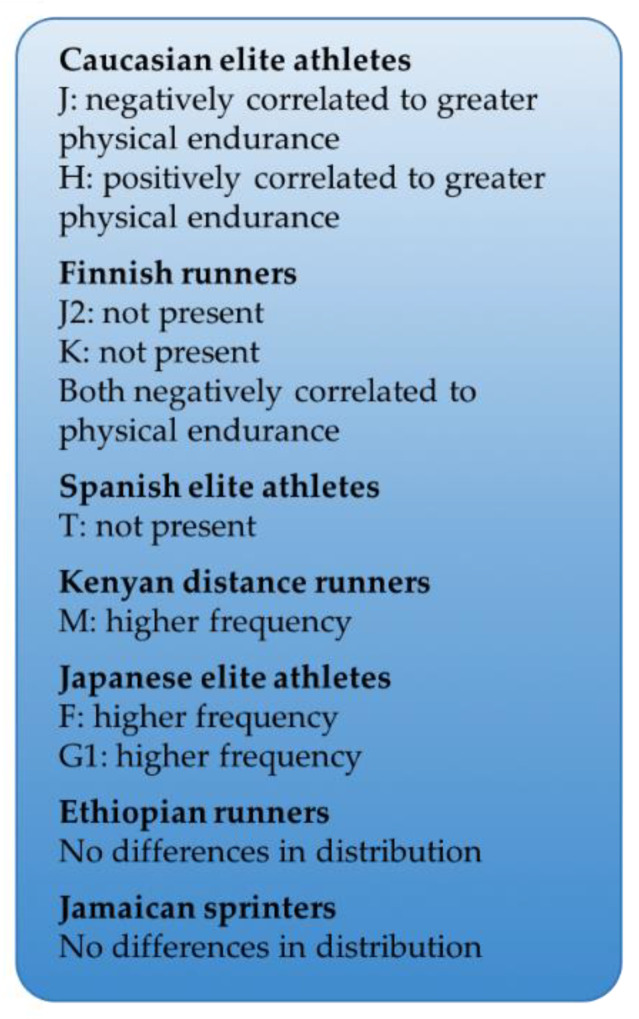
MtDNA haplogroups’ distribution among elite athletes compared to the general populations may partially explain their impact in elite athlete performance.

Since mtDNA encoded proteins are involved in exercise response by regulating mitochondrial metabolism and mass, it is interesting to highlight the possible relationship between mtDNA variants, namely mtDNA haplogroups, and the capacity to achieve elite endurance athlete status. Oxidative phosphorylation (OXPHOS) activity can be modulated by mitochondrial haplogroups, which can impact mitochondrial metabolism and play a role in several physio-pathological conditions [3,25,26]. Some studies have investigated how mtDNA polymorphisms can affect the performance of athletes. In Caucasian athletes, haplogroup J seems to be related to lower VO_2_max, efficiency of electron transport chain (ETC), and a reduction of ROS and ATP production, whereas haplogroup H leads to higher VO_2_max and greater physical endurance during prolonged exercise [3]. Interestingly, a sub-group of the haplogroup J, namely J2 along with the haplogroup K, has never been identified in elite Finnish endurance athletes. These two haplogroups are associated with longevity and are likely to be disadvantageous for endurance sports, as they make OXPHOS less efficient [27]. Similarly, the haplogroup T was found to be negatively associated with elite Spanish endurance athlete status [28]. However, the landscape of mtDNA haplogroup distribution in elite athletes is not homogeneous and differs among different populations. Compared to the general population, an excess of M haplogroups among elite Kenyan athletes [3] and an over-representation of haplogroup F and G1 among elite Japanese athletes [29] have been reported. Conversely, no observed differences in haplogroups distribution have been reported between Ethiopian endurance athletes [29] or among Jamaican athletes (observation according to SNPs analysis) [29] compared to the general populations. These associations between elite athlete status and some haplogroups could suggest that mtDNA haplogroups influence some aspects of exercise performance, likely through direct or indirect changes in the efficiency of OXPHOS and ATP synthesis (Figure 2). However, it must be noted that no direct evidence has been provided to confirm this hypothesis.

## 3. Mitochondrial DNA as a Signaling and Pro-Inflammatory Molecule

### 3.1. Cellular Pathways Triggered by cf-mtDNA

Zhang et al. initially proposed that mtDNA, released in circulation after trauma, is a causative event of sterile inflammation [30]. Subsequent studies have reported that the release of mtDNA in the extracellular space and into the cytoplasm is caused by various stimuli, including stress, trauma, pathogens, and ROS. Several studies have revealed that essentially, the release of mtDNA activates three different pattern recognition receptors (PRRs) and innate immune responses, including cyclic GMP-AMP synthase (cGAS)-stimulator of interferon genes (STING) signaling, Toll-like receptors (TLRs), and inflammasomes (Table 1) [31,32,33].

cGAS, a DNA sensor protein present in both the plasma membrane [45] and cell nucleus [46], recognizes misplaced DNA (cf-mtDNA). cGAS binds to dsDNA, inducing the synthesis of cyclic guanosine monophosphate–adenosine monophosphate (cGAMP) from ATP and GTP, thanks to a conformational change [47,48]. The endoplasmic reticulum (ER)-resident protein acts as a STING and is bound by cGAMP [34,36], then TANK-binding kinase 1 (TBK1) is required for interferon regulatory factor 3 (IRF3) phosphorylation. Once dimerized and translocated into the nucleus, IRF3 stimulates the expression of several pro-inflammatory genes, including type I interferons (IFN-I) [34,35,36]. Apoptotic caspases, which cleave cGAS, can activate this pathway. BAK and BAX permeabilize the mitochondrial outer membrane, and, with caspase inhibition, these pores grow and allow inner membrane herniation and extrusion of mtDNA, which in turn can bind cGAs in the cytosol and trigger an inflammatory response [49].

Hypomethylated or oxidized cf-mtDNA is a potent activator of TLR9, an endosome resident protein, which can recognize nucleic acids and then trigger downstream signaling cascades mediated by the adaptor molecule myeloid differentiation primary response gene 88 (MyD88). MyD88 in turn stimulates mitogen-activated nuclear transcription factor kappa B (NF-kB) and protein kinases (MAPKs), thereby inducing the production of proinflammatory cytokines and chemokines that lead to inflammation [37]. In addition, TLR9-MyD88 signaling can also increase the production of IFN-I through the activation of transcription factor interferon regulatory factor 7 (IRF7) [38].

It has been shown that cf-mtDNA plays a crucial and specific role during NLRP3 inflammasome activation. In mouse macrophages, it has been demonstrated that cf-mtDNA is required for the activation of NLRP3 inflammasome [41]. Interestingly, NLRP3 appears to prefer oxidized cf-mtDNA. Sterile injury or infection can stimulate a cytosolic protein complex formation, including inflammasome effector, sensor, and adaptor. This results in inflammation because of self-cleavage of procaspase 1 and activation of caspase1, able to proteolytically convert immature cytokines, pro-IL-18 and pro-interleukin-1b (pro-IL-1b), into their biologically active forms [42,43].

The inflammatory nature of cf-mtDNA has been confirmed in numerous studies over the last few years. Our group investigated the possible role of cf-mtDNA during “inflamm-aging”, a condition of chronic, low-grade inflammation, typical of senescence and related to the onset of several comorbidities which commonly affect older people [50,51]. We observed an increase of cf-mtDNA during aging, especially after the fifth decade of life, compared with younger subjects, with maximum levels observed in subjects over 90 years old. This increment is associated with high plasma proinflammatory cytokine levels, in particular IL-6, TNF-α, IL-1ra, and RANTES. Thus, our data suggest that plasma cf-mtDNA can contribute to the proinflammatory status observed during inflammaging [52].

### 3.2. Effects of cf-mtDNA on Central Nervous System

Given that mitochondria are abundant in dendrites, nerve terminals, and axons, mitochondrial products can act as DAMPs when they are released into extracellular space. Cell injury induces the entry of cf-mtDNA and other DAMPs into the cerebrospinal fluid (CSF) or the bloodstream for their recognition by the innate immune system, causing a systemic or local response. Cf-mtDNA is considered a potential biomarker, where increased levels indicate physio-pathological conditions of chronic inflammation [52,53,54]. Microglial cells and perivascular macrophages are the only resident immune cells of the brain parenchyma. After the destruction of microenvironment homeostasis, microglia acts to defend the brain, changing its phenotype acquiring pro- or anti-inflammatory function. In particular, the pro-inflammatory function is related to the pathogenesis of neurological disease prompted by inflammation, such as epilepsy, neurodegenerative and demyelinating diseases, and central nervous system trauma. Microglia can recognize DAMPs through the PRRs, detecting stress and cellular damage. Our group has studied ROS production in HMC3 cells, a human microglial cell line, submitted to cf-mtDNA treatment [55,56]. This study highlights an increase in ROS production and the synthesis of proinflammatory cytokines [57]. Furthermore, ROS generation is one of the main factors able to trigger NLRP3 inflammasome activation, which is responsible for cleavage and release of the mature form of some inflammatory cytokines, such as IL-18 and IL-1β.

CSF cf-mtDNA is important in the development of neuro-immunological disorders because it acts as a link between the immune system and brain and has been extensively analyzed in patients affected by both Parkinson’s (PD) and Alzheimer’s disease (AD) [58]. In symptomatic patients with familial and sporadic AD and asymptomatic at-risk individuals, CSF cf-mtDNA concentrations are significantly lower if compared with the concentration measured in healthy age-matched controls [59]. Further, sporadic PD patients show decreased concentrations of CSF cf-mtDNA [60]; although AD and PD are characterized by neuronal death, which normally leads to a massive release of cf-mtDNA, this study shows unexpected low levels of cf-mtDNA. Cf-mtDNA has also been investigated in multiple sclerosis (MS), another neurodegenerative disorder which also induces a strong inflammatory response. Interestingly, patients with MS show higher cf-mtDNA levels, in contrast to what is observed in people affected by PD or AD [61].

## 4. Release, Forms and Detection of cf-mtDNA

### 4.1. Mechanisms of Release and Removal of cf-mtDNA

In the last few years, several studies have proposed the potential mechanisms involved in the release of mtDNA from cells, including either passive or active processes [62]. Passive mtDNA release mechanisms include apoptosis and necrosis. Apoptosis is a programmed process of cell death, characterized by cellular dismantling, leading to the production of apoptotic bodies, containing various cellular components including mtDNA. Since apoptotic bodies can enter circulation, apoptosis seems to be the main source of cf-mtDNA release in plasma [63,64]. In head and neck squamous cell carcinoma and non-small-cell lung cancer cell lines, plasma cf-mtDNA seems to be released not only by apoptosis but also by tumor cell necrosis [63,65].

Under physiological conditions, active regulated mechanisms seem to be responsible for cf-mtDNA release in plasma, such as exosomes and neutrophil extracellular traps (NETs) [66,67,68]. Exosomes (30–140 nm) are a type of extracellular microvesicles that are released into the circulation, involved in multisystemic effects of endurance exercise mediation, because they can transfer several biomolecules, such as vesicles containing mtDNA, from muscle. The exosome marker apoptosis-linked gene 2 interacting protein X (ALIX) is present in skeletal muscle and is depleted after acute endurance exercise, supporting the hypothesis that exosomes are released in response to endurance exercise in muscle [66]. Recent studies suggest that in stimulated human neutrophils, cf-mtDNA results present in NET and, as in a vicious cycle, are also able to induce NET formation through TLR9 signaling [69]. Under experimental conditions, cf-mtDNA release can be promoted by several cell types, including leukocytes, platelets, astrocytes, endothelial cells, and immortalized cell lines [62]. However, physiological source(s) of cf-mtDNA are yet to be identified.

In addition, trauma or hemorrhagic shock has been proven in both human and animal experimental models to be a leading cause of cf-mtDNA release into circulation. In humans, shock activates neutrophils p38 MAPK pathway and TLR9 signaling, triggering matrix metallopeptidase 8 (MMP-8) and 9 (MMP-9) release [70]. MMPs can degrade the basement membrane and all components of the extracellular matrix (ECM). Moreover, MMPs are involved in the activation or release of growth factors, cytokines, chemokines, antibiotic peptides, and other bioactive molecules, regulating several physiological processes such as inflammation, neurite growth, angiogenesis, innate and adaptive immunity, and bone remodeling [71].

There seem to be two major mechanisms involved in cf-mtDNA removal: cf-mtDNA degradation in the blood by circulating DNases and cf-mtDNA uptake by target cells [59]. DNases are enzymes secreted by various organs into different body fluids including blood, able to hydrolyze circulating DNA molecules [72,73]. DNases are triggered by various signals, such as apoptosis in response to bacterial and viral infection [73,74]. Velders et al. highlighted that even a single bout of high-intensity exercise, in healthy people, can enhance circulating DNase activity [73]. Otherwise, cf-mtDNA may be removed via the liver, spleen, or kidney [62,75]. In septic patients with kidney injuries, cf-mtDNA levels are elevated in urine but not in plasma, supporting the idea that urine is a possible mechanism of cf-mtDNA elimination [76].

### 4.2. Detection of cf-mtDNA in Plasma

As described above, cf-mtDNA, released through passive or active mechanisms, can be found in different physiological forms in circulation, as whole mitochondria, packaged in vesicles bound to protein, naked or even fragmented. However, we focused on the quantification of cf-mtDNA in plasma, as this is the most frequently used method of analysis in samples from people who practice sport. For a more detailed discussion on different body fluids, we suggest the systematic review of Trumpff et al. [62]. Briefly, pre-analytical processes are crucial for quantifying cf-mtDNA in plasma and their levels are strictly dependent on the centrifugation conditions in terms of time and speed. After isolation of the plasma from blood cellular fraction, plasma specimens must be further centrifuged at 5000× *g*, 10 min to remove platelets, which contain high amounts of mtDNA copies, and large vesicular bodies. Then, the supernatant can be aliquoted and stored at −80 °C for the quantification of cf-mtDNA. Additional centrifugations permit the elimination of progressively different forms on mtDNA bound to a membrane (including whole mitochondria and extravescicles of different sizes) or protein, until only naked DNA is obtained. Total DNA from plasma can be extracted using a spin-column based commercial kit [77] and quantified through quantitative PCR-based techniques, such as the quantitative Real-time PCR [52,53,54,78] and the droplet digital PCR [77]. Real-time PCR requires the use of a reference standard curve whereas ddPCR does not. With both methods, the use of a “no template” and a “positive control” is mandatory in each plate. It would be preferable to perform a simultaneous quantification of cf-mtDNA and cf-nDNA to identify if nuclear or mitochondrial DNA is preferentially released. Levels of cf-mtDNA could be reported as copies or nanograms of mtDNA per unit of volume (general copies per mL) of plasma.

## 5. The Effects of Trauma/Injuries on cf-mtDNA Release

### 5.1. The Effects of Traumatic Events on cf-mtDNA Release

Several studies suggest that cf-mtDNA has an important role in activating three main cellular signals, such as the cGAS-STING pathway, TLR9, and the inflammasome. Therefore, cf-mtDNA can be considered an important inflammatory driver involved in many human diseases [8], including cancer [79,80], trauma, stroke [81], pulmonary embolism, liver failure [82], and sepsis [83]. Several clinical studies have highlighted that injury severity, outcome, and cf-mtDNA levels are correlated. These observations lead to the notion that levels of cf-mtDNA could be a new biological marker of injury severity for critically ill patients [7]. Cf-mtDNA present in NETs is highly interferogenic, and its extrusion by neutrophils contributes to the onset of inflammatory autoimmune diseases, such as systemic lupus erythematosus, not only in mice but also in humans [5,84,85]. Ox-mtDNA activates STING and TLR9 signaling stimulating proinflammatory cytokines and IFN-I production, proven to promote lupus-like disease development in mice [5].

In addition, clinical studies with trauma patients have demonstrated that cf-mtDNA levels can be considered an independent predictive factor for the improvement of post-traumatic systemic inflammatory response [4,7].

Furthermore, cf-mtDNA is involved in the pathogenic progression of nonalcoholic steatohepatitis (NASH), one of the most common liver diseases, activating TLR9 signaling. Supporting this data, high levels of cf-mtDNA have been found in plasma from both mice and patients with NASH [39].

High levels of cf-mtDNA in plasma and body fluids are also detected in patients with inflammatory disorders. For example, cf-mtDNA increased levels in some fluids, such as synovial fluid and plasma, were observed in rheumatoid arthritis patients compared to healthy volunteers [86]. Data from our group reveal that the presence of cf-mtDNA and other DAMPs of mitochondrial origin in the peripheral blood of patients with HIV infection indicate that these molecules play a central role in typical inflammation associated with HIV infection [87]. Cf-mtDNA can also activate TLR9 signaling in cardiomyocytes, promoting the progression of cardiovascular disorders [88].

Cf-mtDNA also plays a central role in the pathogenesis of ocular diseases. It has been shown that cf-mtDNA is involved in the pathogenesis of age-related macular degeneration (AMD), leading to blindness, and is characterized by the death of retinal pigmented epithelium (RPE) [80]. Cf-mtDNA is damaged in the retinas of patients with AMD, and its capacity for repair is decreased. Several studies suggest that AMD development is due to NLRP3 inflammasome activation, which creates an inflammatory microenvironment as a result of cf-mtDNA accumulation in aged RPE cells [44,89].

Clinical studies report high cf-mtDNA levels in patients with acute kidney injury (AKI), a cluster of syndromes that shows a rapid decline in renal function within days, or in some cases within hours [90]. Therefore, cf-mtDNA seems to be related to AKI prognosis and severity and may be considered a predictive factor for this disease. Cf-mtDNA plays an indispensable role in the stimulation of innate immune signaling pathways, consequently, mitochondrial dysfunctions cause oxidative stress [91], inflammatory cytokine accumulation, and apoptosis leading to tubular injury [92].

Emerging evidence suggests that DAMPs, such as cf-mtDNA, contribute to the evolution of multiple organ dysfunction syndromes considered a consequence of severe injury that could be potentially fatal [93]. Thus, patients with high cf-mtDNA levels have a greater relative risk of mortality [94].

Critically ill patients show several alterations, such as cellular injury and programmed necrosis, causing an active ejection of cf-mtDNA into plasma by leukocytes. An association between cf-mtDNA levels in plasma and the presentation of acute respiratory distress syndrome (ARDS) has been identified. Trauma and sepsis cause 54% of ARDS cases [95].

### 5.2. Injuries/Trauma in Sports

A significant increase in cf-mtDNA has been reported in the plasma of trauma patients, and the levels of cf-mtDNA are a useful parameter in post-traumatic prognosis. In particular, it has been demonstrated that cf-mtDNA levels are statistically significantly higher in trauma patients who died when compared to survivors [96]. One hypothesis is that damaged tissues induce the release of cf-DNA, including cf-mtDNA, into the bloodstream. Therefore, these data reveal a positive correlation between cf-mtDNA levels in plasma and injury severity.

Another important factor to consider is the difference between young and elderly trauma patients. Following trauma, young patients show an increase in NETs production, without any ex vivo stimulation, that is not found in elderly patients. Moreover, elderly trauma patients show higher levels of cf-mtDNA than younger patients, probably because the elderly are more prone to necrosis after the same type of trauma because of reduced tissue perfusion. It may also be related to the reduction in cf-mtDNA clearance efficiency in the elderly [69].

Among teenagers and young adults, the principal cause of disability following trauma is spinal cord injury (SCI). Traumatic events cause ischemia or neuroinflammation which, in turn, can promote SCI [97]. SCI patients show significantly higher cf-mtDNA levels compared to controls [98]; therefore, we can assume that cf-mtDNA levels are correlated with the severity of the injury and with the presence of post-traumatic complications [99]. Rotational and/or linear forces provoke concussions when transmitted to the brain. This situation is characterized by microscopic axonal injury accompanied by a cascade of metabolic, ionic, and pathophysiological events [100]. Before a possible brain recovery, a second injury occurs inducing more significant cognitive deficits and harmful cellular metabolic changes. Consequently, the concussed brain shows a decline in physiological neural activation [101]. Competitive athletes are often affected by cardiovascular alterations and the most common is hypertension. For example, American football players show an increase in blood pressure and hypertension because, during football matches, they are prone to musculoskeletal trauma, which induces DAMPs release, leading to innate immune system activation. Cf-mtDNA activates TLR9, causing endothelial dysfunction and hypertension. Therefore, repeated injury leads to elevated cf-mtDNA levels inducing a significant activation of TLR9 signaling. Consequently, football players show increased vascular tone, impaired endothelium-dependent vasodilation, and hypertension [40]. Skeletal muscle damage depends on both metabolic and mechanical factors and is caused by intense and strenuous exercise. Prolonged exertion, such as ultra-endurance events and marathon running, has been associated with negative cardiovascular alterations [102] and other markers of damage, including hypertension induced by exercise [103]. Repetitive concussive and sub-concussive events to the brain lead to cumulative neurologic consequences causing chronic traumatic encephalopathy. In general, repetitive trauma is responsible for depression and dementia-like syndromes in American football players. Regarding the crucial role of cf-mtDNA and high mobility group box (HMGB) in neuroinflammation because of traumatic brain injury [104], it has been hypothesized these DAMPs can be mediators not only in hypertension but also in other conditions defined by chronic inflammation among American footballers.

## 6. The Effects of Exercise on cf-mtDNA Release

Despite the interest in cf-mtDNA as a possible contributor to inflammation, few studies are evaluating the effects of exercise on this marker. Available studies are listed in Table 2, which also includes subjects’ descriptions, age, type of exercise to which they were subjected, and the main effects on cf-mtDNA.

Beiter et al. analyzed the cf-mtDNA levels in nine well-trained men after an incremental test on a motorized treadmill: the running velocity was increased every 3 min until subjects’ exhaustion [105]. Cf-mtDNA measured before, immediately at exhaustion, and after 30 min remained unchanged. In the study performed by Helmig et al. five healthy, physically active men who usually trained for more than 3 h per week were subjected to the same conditions on a treadmill as those reported by previous studies [106]. Similarly, subjects’ plasma cf-mtDNA remained unchanged before, immediately at exhaustion, and after 10, 30, and 90 min (at rest). After plasma centrifugation with two different protocols, Helmig et al. also quantified cf-mtDNA in the supernatant and the pellet (treated or not with DNAse I) to ascertain the possible contribution of mtDNA contained in extravescicles (EVs). Again, they could not determine significant changes in mtDNA in any fraction. In our previous study, for two consecutive years, we analyzed the plasma concentration of cf-mtDNA in 12 professional male volleyball players (throughout seasonal training) and a control group of 20 age-matched non-athlete male volunteers [54]. We observed that controls have higher levels of cf-mtDNA compared to athletes, in which this marker decreases during the first season but not in the successive season, even if a trend was appreciable. Shockett et al. evaluated the response of seven healthy moderately-trained men to a ninety min graded exercise treadmill test with a progressive increment of grade and velocity [107]. Blood was collected at baseline, during the exercise (at 18 and 54 min), at the end of the exercise (90 min), and after 40 min of resting. Results were compared to those obtained 1 month later in the same subjects (the control group) rested in a seated position with no exercise. During exercise, levels of cf-mtDNA seem to decrease and return to baseline values after 40 min of rest. Moreover, at 54 and 90 min, they were significantly lower compared to controls. It is notable that, unlike other studies in which mtDNA was quantified by the Real-time PCR technique, cf-mtDNA was measured by PCR and subsequent agarose gel electrophoresis image analysis. Stawski et al. evaluated the release of cf-mtDNA before and after an exhaustive treadmill, without increment of grade or velocity [108]. Eleven averagely-trained men were subjected to three very intense treadmill sessions until exhaustion, every 3 days, to ascertain the effects of both acute and repetitive exercise. Each session determined an increase in cf-mtDNA (statistical significance was observed following the second and third sessions only), pointing to the hypothesis that cf-mtDNA probably requires more than one exhaustive exercise to increase. The authors explain that a further difference could be ascribed to the intensity of the exercise. Moreover, cf-mtDNA levels were found to decrease over the study before each exercise session. Interestingly, Stawski performed a qualitative analysis of mtDNA in 2019 (not reported in Table 2) using the same samples. This study reported that the integrity of mtDNA fragments did not change in response to exercise, despite that they should be more sensitive to reactive oxygen species due to a lack of protective proteins and an efficient DNA damage repair mechanism [110].

The study performed on scuba divers, by Blatteau et al., is of interest because it is considered underwater swimming [113]. During a dive, the environmental conditions can change rapidly, causing an increased physical activity and a higher production of free radicals [111], which can damage mitochondria favoring the release of mtDNA. Scuba diving is not usually considered a strenuous sport, since the required exertion is typically mild [112], but the unforeseeable environmental conditions can cause physiological (cardiovascular system, ears, and lungs) and emotional stress. Comparing eight scuba divers with 22 non-divers, the authors found that divers have less cf-mtDNA than non-divers. Thus, it seems that also an underwater activity, like other regular physical activities, could modulate cf-mtDNA.

Returning to acute exercise, Hummel et al. enrolled 20 healthy males and subjected them to an incremental exhaustive treadmill. Immediately after the exercise cf-mtDNA was found to increase and then returned to baseline values after 15 and 30 min of rest [109]. Similarly, Ohlsson et al. analyzed the release of cf-mtDNA in four females and four males, before and during a high-intensity incremental ergometer cycle test (at submaximal- and maximum load), and after 30 and 90 min of resting [114]. Cf-mtDNA increased progressively, reaching a statistical significance at 30 and 90 min of rest. Finally, we mention a study performed by Walczak et al. including 14 men with type 1 diabetes mellitus (T1DM) compared to 11 healthy controls (not included in Table 2) [115]. Enrolled subjects performed exercise on a treadmill until exhaustion at a constant speed. Blood samples were collected pre- and post-exercise. In both groups, cf-mtDNA was comparable and no changes were observed. Despite not being strictly associated with a specific exercise, we consider it useful to cite our previous study on the effects of whole-body cryotherapy (WBC) on cf-mtDNA in non-professional runners and cyclists, in which we found that three one-day WBC treatments did not affect cf-mtDNA levels in athletes during the period of training [77].

It must be stressed that all studies reported in Table 2 have a clear limitation in sample size. Considering the studies in which participants performed a single treadmill or ergometer acute exercise, participants were well trained in only two of them [105,106], while in the remaining studies, participants were average or moderately trained. It cannot be ruled out that the discrepancies among studies may be ascribed to participants’ characteristics. One study performed a semiquantitative–PCR, rendering a comparison with others studies difficult [107]. As also discussed by Trumpff at al. differences in protocols for plasma processing and cf-mtDNA extraction and quantification affect results. The application of standardized methods will increase our ability to understand the dynamics of cf-mtDNA in different physio-pathological conditions [62]. Finally, three studies showed that participants playing volleyball, diving or subject to repetitive exercises are characterized by a decrease of cf-mtDNA. This may suggest that cf-mtDNA decrease associated with sports could, at least in part, contribute to the typical anti-inflammatory effects of regular exercise.

## 7. Conclusions

Although studies concerning the effects of physical activity on cf-mtDNA are still largely preliminary, they pave the way for further studies to better understand not only how exercise modulates cf-mtDNA but also how mtDNA contributes to inflammation in people who perform different types of physical activity, with particular regards to contact sports. In our opinion, there are two fundamental questions that remain unanswered and need further investigation: (1) the effects of mtDNA as pro-inflammatory molecules in activities that involve physical contact and chronic trauma and (2) the mechanisms involved in the release and the clearance of mtDNA during acute or repetitive/regular exercise and at a resting phase.

On one hand, the increase of mtDNA after acute strenuous exercise, or acute exercise sessions, is consistent with a release from skeletal muscle cells due to hypoxia or tissue damage. On the other hand, it is unlikely that the release of cf-mtDNA after acute time-limited exercise, such as a single heavy acute bout, is due to apoptosis or necrosis. More probably, living damaged cells might be responsible for its acute increase.

As described, cf-mtDNA could be released during NET formation by granulocytes or tissue resident cells (i.e., macrophages, mast cells). It remains to ascertain if moderate exercise would be more compatible with a passive lower release of mtDNA. Additionally, the clearance of cf-mtDNA is far from being understood.

**Figure 3:**
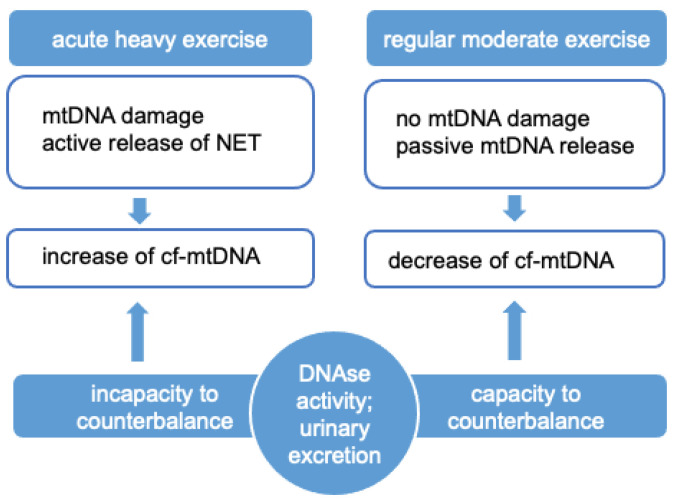
Proposed mechanism for the release and removal of cf-mtDNA after physical activity.

In conclusion, preliminary findings on the effects of exercise on cf-mtDNA seem to indicate that acute, heavy exercise may lead not only to cellular mtDNA damage and but also to its augmented release in circulation, probably by active mechanisms related to a cell injury. On the other hand, regular, moderate exercise seems to favor the removal of mtDNA through mechanisms that have not yet been fully elucidated. One proposed mechanism is the enhancing of circulating DNase activity mediated by acute heavy exercise. However, the role of the DNase enzyme in regular physical activity has not yet been established. It may be argued that the DNase effect may not be able to counterbalance the high release of mtDNA during heavy exercise but is able to mediate the levels released during regular moderate physical activity. Moreover, the ratio of cf-mtDNA loss by urinary excretion in relation to different types of exercise should be determined (Figure 3). This speculation leads back to the importance of discriminating the contribution of different forms among total cf-mtDNA, as only certain forms of DNA (e.g., DNA not contained in vesicles) can be enzymatically digested. Despite the fact that physical activity characterized by repeated trauma/injuries, such as contact sports, can lead to the potential increase in mtDNA, regular exercise is the only factor currently known able to reduce mtDNA in systemic circulation, again underlining the beneficial effects of exercise as a tool for acute and chronic disease prevention.

## Figures and Tables

**Table 1:** Pathways activated by mtDNA release on different cell types.

Pathway	Effects	Cell Type	References
cGAS-STING signaling	IFN-stimulated genes induction	Neutrophils Macrophages	[5,34,35,36]
TLR-9	Proinflammatory cytokines and chemokines production IFN-I production	Hepatocytes Cardiomyocytes Leukocytes Neutrophils Endothelial cells	[18,37,38,39,40]
NLRP3 inflammasome	IL-1β and IL-18 activation	Macrophages RPE cells	[41,42,43,44]

**Table 2:** Effects of exercise on cf-mtDNA.

References	Subjects	Age * (Years)	Type of Exercise	Effects on cf-mtDNA
[105]	*n* = 9 well-trained men	29.3 ± 8.5	Incremental exhaustive treadmill	=before, immediately after and after 30 min of rest
[106]	*n* = 5 healthy, physically active men	26.8 ± 2.2	Incremental exhaustive treadmill	=before, immediately after, 10, 30 and 90 min of rest
[51]	*n* = 12 male volleyball players; *n* = 20 non-athletes	27.5 ± 3.9; 29.5 ± 4.5	Volleyball	↓ in the first in-season training period and = in the second in-season training period.
[107]	*n* = 7 healthy moderately trained men	22.4 ± 1.2	Ninety min treadmill	↓ during exercise in comparison to control group (same subjects in seated position)
[108]	*n* = 12 average-trained men	34.0 ± 5.2	Exhaustive treadmill	↑ post-exercise; ↓ pre-exercise over the study period
[109]	*n* = 8 free divers; *n* = 22 non-divers	36.9 ± 9.6; 52.3 ± 14.5	Diving	divers have less mtDNA than non-divers
[110]	*n* = 20 healthy males	23.3 ± 3.8	Incremental exhaustive treadmill	↑ post-exercise; ↓ after 15 and 30 min of rest
[111]	*n* = 8 healthy volunteers (4 women and 4 men)	38.6 ± 14.4	Incremental exhaustive ergometer cycle	↑ during exercise, compared to baseline values and after 30 and 90 min of rest
[112]	*n* = 14 men with T1DM **; *n* = 11 healthy controls	29.3 ± 5.3; 34.0 ± 5.2	Exhaustive treadmill run	=before and after exercise in both groups

* Mean ± SD, ** T1DM = type 1 diabetes mellitus.

## Data Availability

Not applicable.
